# Financial Incentive Required for Pharmacy Students to Accept a Post-Graduation Position in Rural and Undesirable Pharmacy Settings

**DOI:** 10.3390/pharmacy7030109

**Published:** 2019-08-06

**Authors:** Erin Ulrich, Jonathan Hurdelbrink, Jason Perepelkin, Kelli Welter

**Affiliations:** 1 College of Pharmacy and Health Sciences, Drake University, Des Moines, IA 50311, USA; 2 College of Pharmacy and Nutrition, University of Sakatchewan, Saskatoon, SK S7N 5C9, Canada

**Keywords:** pharmacy student, rural, rural pharmacy, job preference, economic valuation

## Abstract

**Background:** It has been estimated that in 2018, 20% of pharmacy students were unemployed following graduation. However, many pharmacy positions go vacant each year, with the majority of these positions existing in rural areas. **Methods:** Pharmacy students completed a one-time, anonymous, online questionnaire. Measures of interest included: subject characteristics and preference in a variety job offers. Discrete Choice Experiment methodology of questionnaire design was used and Conditional Logit models were conducted to analyze the data to determine the financial incentive required for pharmacy students to take a post-graduate job with particular traits. **Conclusions:** A total of 283 students completed questionnaires from Iowa, North Dakota, South Dakota, Saskatchewan, and Manitoba. The majority of subjects were female, P3 students, and from a non-rural hometown. American students would need to be paid an additional $18,738 in salary to practice in a rural area, while Canadian students would require an additional $17,156. Canadian respondents would require an additional $7125 in salary to work in a community pharmacy with a low level of patient interaction compared to a community position with a large amount of patient interaction. Overall, pharmacy student preferences in post-graduation job attributes vary significantly between states and provinces.

## 1. Introduction

It has been estimated that in 2018, 20% of pharmacy students were unemployed following graduation [1,2]. Many relate this to the increased number of pharmacy schools producing more graduates than the available positions [2,3,4,5]. An alternative explanation proposed is that the pharmacy positions have not grown as fast as predicted because the profession is shifting from dispensing activities to patient care services slower than planned [3,5]. However, it has been reported that there remains between a 5.5%–6.0% growth projection in pharmacy careers in the next 10 years [6,7]. There are many unfilled pharmacy positions each year, with the majority of these positions existing in rural areas or certain states [8,9]. While new graduates should be filling these vacancies, which are expected to grow as pharmacists in these areas retire, they are not doing so as they may view these settings as relatively less desirable; in other words, the majority of students may be applying and competing for the limited positions that they deem more desirable. It has been suggested that increasing salary could be used to incentivize graduating pharmacy students to practice in these high-need areas due to their significant amount of student loan debt; specifically, 2018 American pharmacy students attending public schools have on average taken out $137,356 in loans whereas those attending private schools have on average taken out $193,296 in loans [10,11].

Although evidence of the effectiveness of such financial incentive programs for pharmacy students is limited, previous studies have identified the positive effect they have in both the recruitment and retention of other healthcare providers to underserved areas. For example, greater salaries, financial aid, and/or loan reimbursement have been found to effectively recruit more physicians in West Virginia to practice in rural areas [12] and graduating health professionals in New Mexico to choose rural areas as their first practice location [13]. In order to be effective, however, these incentives must be sufficient in magnitude and known to students/providers [13]. In addition, financial incentives alone may not be sufficient to successfully recruit healthcare providers to practice in these areas. Specifically, additional factors such as the availability of professional opportunities, the desirability of the rural locations [13], and exposure to family practice and rural health during one’s schooling [14] have been found to impact one’s decision over practice location. As a result, many believe that incentive programs must address several of these factors in order to be truly effective.

To help address this imbalance, the U.S. program Medicare has created the term Health Professional Shortage Areas (HPSAs). If a medical professional practices in a HPSA for four years, they are eligible for student loan repayment, scholarship, and additional public health service programs. The National Health Service Corp (NHSC) Loan Repayment Program funds healthcare professionals up to $160,000 over four years for taking a post-graduate position in a medically underserved area [15]. The NHSC Loan Repayment Program may include pharmacists depending on the location. Many states have established alternative ways, such as PRIMECARRE in Iowa, for pharmacists to receive financial incentives for practicing in their rural areas [16]. Through this program, pharmacists can receive up to $50,000 for loan repayment for providing services in specified health professional shortages area (HPSA) per HRSA guidelines. Pharmacists must work in an HPSA-eligible site for a minimum two years of full-time work OR four years of part-time work. It is common for these alternative loan repayment programs to provide around $50,000. Although generous, it is significantly lower than the average amount of student loans taken out by pharmacy students as discussed above. 

Due to the complexity of the supply/demand situation for American graduates, it is important to include Canadian pharmacy students in this conversation. Although Canadian pharmacy students take out significantly fewer student loans than their Southern neighbors do, Schools of Pharmacy have recently converted from a BS to Pharm D degree. The Canadian pharmacy profession is also embracing the transition from dispensing to patient care services and the evolving scope of practice [17]. In addition, Canada also experiences health disparities and issues with access to health care in their rural areas [18]. With this information in mind, it is critical that Canadian pharmacy students be included in workforce research to prevent the same dilemma and hardships for future graduates. 

By understanding what financial incentives are necessary for students to work in a variety of settings, employers would also know how much additional salary would be necessary to attract students to fill their open, less desirable positions. The objective of this study was to determine required financial incentive to encourage pharmacy students to work post-graduation in different practice and geographic settings in NABP/AACP District 5 (Iowa, North Dakota, South Dakota, Nebraska, Minnesota, Saskatchewan, and Manitoba). 

## 2. Materials and Methods

### 2.1. Inclusion Criteria

Individuals were asked to participate in this study if they were:

(1) Enrolled as a pharmacy student at the time of data collection, and
(2) Attending one of the nine pharmacy schools in the NABP/AACP District V: The University of Iowa College of Pharmacy, Drake University College of Pharmacy and Health Sciences, University of Minnesota College of Pharmacy (both Twin Cities and Duluth locations), University of Nebraska Medical Center College of Pharmacy, Creighton University School of Pharmacy and Health Professions, South Dakota State University College of Pharmacy and Allied Health Professionals, North Dakota State University School of Pharmacy, University of Saskatchewan College of Pharmacy and Nutrition, or University of Manitoba College of Pharmacy.

### 2.2. Sampling

Investigators contacted schools of pharmacy to determine interest and ability to participate. If interested, schools emailed out an IRB-approved recruitment email to students in their pharmacy program. The email included a link to a one-time, anonymous Qualtrics questionnaire. 

### 2.3. Data Collection

When subjects clicked the provided link, they were directed to an informed consent page. Informed consent was assumed if students clicked to continue onto the questionnaire. Reminder emails were encouraged to be sent two weeks after initial email. Three out of the six participating schools did not send reminder emails to their schools due to institutional policies on frequency of emails sent to their students. Data collection occurred from 1 March 2018–10 April 2018.

### 2.4. Questionnaire Design: Discrete Choice Experiment and Economic Valuation

This survey methodology, referred to as a Discrete Choice Experiment (DCE), requires respondents to select their preferred job offer from a set, or ‘bundle,’ of four hypothetical options. These options differ based on varying attributes such as salary and geographical location, so the respondent’s choice effectively reflects how they value these attributes relative to one another. Based on these responses and the variation between offers, empirical analyses allow investigators to estimate the amount that students are willing to pay to work in a job with that particular attribute. This section provides background on how the job attributes included in the questionnaire bundles were selected and operationalized.

In order to identify which job qualities and attributes were most desirable to current pharmacy students, third year pharmacy students at Drake University were surveyed utilizing an online, anonymous Qualtrics questionnaire. Approval was obtained from Drake University Institutional Review Board. Informed consent was obtained prior to access to the one-item survey. From a list of 15 job attributes, respondents were asked to select up to five job characteristics they are looking for in a post-graduation job. Of the 103 students asked to participate, 89 responses were obtained (86.4% response rate). Because student pharmacists attending a private school may differ in their career preferences to students at a public institution, intergroup comparison analyses was conducted between Drake University students and the University of Iowa students by determining any significant differences between their preference estimates. 

The five most common characteristics that students prioritize when considering future job offers were geographic location (n = 67, 75%), salary/loan repayment opportunities (n = 52, 58%), level of patient interaction (n = 48, 54%), time to promotion (n = 45, 51%), and interaction with health care team (n = 30, 34%). Investigators then met with students and practicing pharmacists to determine how to operationalize these top five job attributes. One student from each year in pharmacy school was invited to attend a focus group. The student research assistant invited students who would be willing and available to assist. Investigators then used a cognitive interviewing approach to walk students through each attribute to determine how students operationalize these variables. Table 1 outlines the final operationalization of the attributes that were included in the job offers. 

Geographic location. In the United States, urban/rural is defined differently than in Canada. In addition, there are many more categories of city size in the US. To keep consistency between states and provinces and maintain simplicity to prevent response burden, the US Census definitions of urban (>50,000), urban fringe/clusters (2500–25,000), and rural (<2500) were utilized [18]. A student assistant then reached out to students at a college of pharmacy at each school to have those students select towns that best represent the operationalization of urban, urban fringe and rural for their state/province. A map of these selected towns was provided with the job offers. 

Salary. Annual salaries were collected from a variety of job postings. Many job postings in the US were in annual salary, while Canadian jobs were commonly presented in hourly pay. Three amounts were then selected within this range to be placed in the job offers as annual salary for the US questionnaires and hourly pay for the Canadian questionnaires. 

Loan repayment. The cost of higher education in Canada is significantly lower than in the United States. Due to the nature of student loans varying between the United States and Canada, student loan repayment options were only included in the American questionnaires. This job attribute operationalization was modeled after the HPSA health professional incentive program. These percentages reflect the share of one’s student loans that would be paid off in 5 years. 

Level of patient interaction/interaction with healthcare team. From meetings with students and pharmacists, these two job attributes were combined during the operationalization process and retitled to ‘Job Setting.’ The two pharmacy settings were hospital and community, and the amounts of patient interaction within each of these two settings were set to 0%, 30%, and 70%. 

Job Outlook/Time to promotion. From reviewing common community and hospital positions, this attribute was operationalized into 1 year, 3 years, and 5 years.

**Figure 1:**
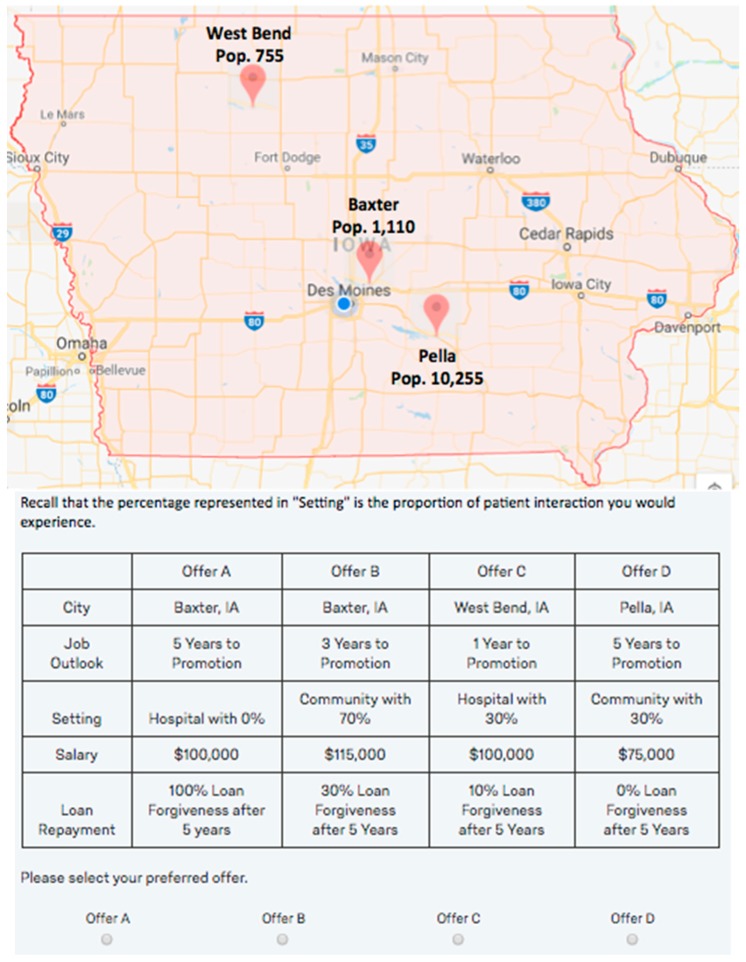
Example of job scenario bundle question.

For each job offer, job attributes were assigned randomly. Each survey includes four unique bundles, meaning that each individual student effectively sees 16 unique job offers. Figure 1 illustrates one such bundle included in the Iowa questionnaire.

### 2.5. Measures of Interest

Questionnaire items include subject characteristics (gender, year in pharmacy school, and size of hometown) and four job offer bundles. 

### 2.6. Statistical Analyses

Frequencies and descriptive statistics were calculated for all variables. Conditional Logit models were estimated using Stata SE 15.1 (College Station, TX, USA) to identify the effect of each attribute on the offer’s likelihood of being selected/preferred:

*Choice*_i,j_ = β_0_ + β_1_ × *Rural*_j_ + β_2_ × *Fringe*_j_ + β_3_ × *Promotion*_j_ + β_4_ × *Comm30*_j_ + β_5_ × *Hosp0*_j_ + β_6_ × *Hosp30*_j_ + β_7_ × *Salary*_j_ + β_8_ × *Loan*_j_ + I_i_ + ε

In this equation, *Choice* simply equals 1 if survey respondent i preferred job offer j in its respective bundle and 0 if he/she did not; in this way, each β reflects how each characteristic influences the likelihood of the offer being preferred, meaning that it similarly reflects how the respondent values each characteristic. *Rural* and *Fringe* similarly equal 1 if offer j included the chosen ‘rural’ or ‘fringe’ town, respectively, and 0 if it does not; *Urban Fringe* is intentionally omitted so that these parameter estimates reflect how respondents value rural and fringe towns relative to an urban fringe town, respectively. *Promotion* equals the number of years from offer j in which the respondent could expect to be promoted to manager. *Comm30*, *Hosp0*, and *Hosp30* equal 1 if offer j included a community setting with 30% of one’s time spent with patients, a hospital setting with 0% of one’s time spent with patients and a hospital setting with 30% of one’s time spent with patients, and 0 if it does not. Similar to the earlier discussion regarding towns, the ‘job setting’ of community pharmacy with 70% of time spent with patients is also intentionally omitted so that these parameter estimates depict how respondents value those respective settings relative to this one. *Salary* equals the proposed salary from offer j measured in thousands of dollars, with Canadian survey results being converted to annual salary for consistent interpretation. *Loan* equals the percentage of one’s loans that would be repaid after five years according to offer j (which is again omitted from Canadian analyses); it is specifically coded as percentage points, meaning that *Loan* ranges from 0 to 100 and β_8_ reflects the effect of a one percentage point increase in loan reimbursement over five years. Lastly, ‘I’ represents individual fixed effects that are included to capture the time-invariant characteristics of each respondent, such as their socioeconomic status or amount of student loans held, that are not otherwise included. 

While the results from these specific analyses are omitted for brevity, they are necessary in calculating the willingness to pay estimates for each job attribute. Willingness to pay (WTP) amounts are calculated by simply dividing each characteristic’s respective β estimate by β_7_, the parameter estimate for *Salary*. The rationale behind this is that, for example, β_1_ represents how respondents value working in a rural town (relative to an urban fringe town) and β_7_ represents how respondents value an additional thousand dollars in salary. Dividing β_1_ by β_7_ therefore yields the amount of salary the respondent would be willing to sacrifice (or, if negative, how much additional salary he/she would require) to work in a rural town instead of an urban fringe town. For ease of interpretation, Canadian hourly pay was converted to annual salary.

## 3. Results

Due to the inherent differences of students who attend a private school versus a public school, it is important to determine if significant differences between these groups exist. Knowing this information is necessary in order to confirm if the original preferences selected by Drake University students to be a part of the questionnaire were appropriate for the remainder of the participating students attending a public institution. Although respondents are not explicitly asked about the university they attend, we were able to identify the students from the two schools by the date of questionnaire completion. Drake University pharmacy students took the online questionnaire a month prior to the University of Iowa pharmacy students. In addition, past research has found that pharmacy students at large, public institutions have the same job traits as students at a private college [19].

A total of 283 students completed questionnaires from the following six colleges of pharmacies who agreed to participate: University of Iowa College of Pharmacy (n = 26), Drake University College of Pharmacy and Health Sciences (n = 71), South Dakota State University College of Pharmacy and Allied Health Professionals (n = 79), North Dakota State University School of Pharmacy (n = 37), University of Saskatchewan College of Pharmacy and Nutrition (n = 22), and University of Manitoba College of Pharmacy (n = 48). Colleges of pharmacies in Nebraska and Minnesota opted out of participation. 

Table 2 displays the subject characteristics of the total sample and each participating school. The majority of respondents were female, P3 students, and from a hometown with a population greater than 2500.

The results in Table 3 and Table 4 present the Unites States and Canadian WTP estimates, respectively. For comparison, the Canadian results have been converted to USD, but the initial estimates still measured in CAD can be found in Table S1 (Supplementary Materials). As can be seen in these tables, WTP estimates may be either positive or negative based on the desirability of its respective attribute. Specifically, a positive WTP amount reflects a desirable attribute that an individual would pay (in the form of accepting a lower salary) to obtain, while a negative WTP amount reflects a relatively undesirable characteristic that graduates would need to be compensated for by means of a higher salary in order to consider. The omitted level of each job attribute is the reference group. Finally, these tables show all WTP estimates but only those that are statistically significant are discussed below.

### 3.1. United States Midwest

American students would need to be paid and additional $18,738 to practice in a rural area relative to an urban area. When stratifying this result by state we see that this result is strongly driven by respondents from Iowa and South Dakota (as those results for North Dakota respondents are statistically insignificant). Similarly, American students would require an additional $12,395 in salary to compensate them for working in a fringe area instead of an urban area, with these results again being primarily driven by respondents in South Dakota. 

American respondents would need to be compensated an additional $2178 for each year they are expected to work before being promoted. Students would need to be compensated with an additional $17,023 in salary to at least consider selecting a hospital position with no patient interaction. However, students would be willing to sacrifice $8544 in annual salary to work in a hospital setting with 30% patient interaction. Lastly, as expected, loan repayment is found to be desirable as well, with students being significantly willing to sacrifice $654 dollars in annual salary for each and every percentage point of loan repayment (i.e., 1%, 2%, etc.) after 5 years. 

### 3.2. Central Canada

Like their American counterparts, Canadian pharmacy students would need to be paid an additional $17,156 and $5198 (in USD for comparison) to work in rural and fringe areas respectively. The number of years before promotion is also found to be undesirable, with students requiring an additional $2149 dollars in annual salary for each year they must work before being promoted to manager. Due to an error made when updating the questionnaire, students in Manitoba were asked to consider some job offers that would require them to work in a community setting without spending any time with patients. As expected, this is a significantly undesirable job setting, with those students requiring roughly $22,734 in additional salary to consider working there as opposed to a community setting with 70% of one’s time spent interacting with patients. In addition, its inclusion is unlikely to influence our overall results despite slightly reducing the power of our analyses since each respondent is only truly evaluating 13 offers (instead of the full 16) after ignoring the three strictly inferior offers with this particular setting. Canadian respondents would require an additional $5277 in salary to work in a community pharmacy with 30% of their time interacting with patients as opposed to one in which they spend 70% of their time with patients. Lastly, Canadian students would require $10,410 in additional salary to work in a hospital setting without any patient interaction (relative to a community setting with 70% interaction) while the statistically insignificant results in the last row imply that they are indifferent between working in a hospital setting with 30% patient interaction and a community setting with 70% patient interaction.

## 4. Discussion

### 4.1. United States

The $18,738 in additional salary required for the American Midwestern pharmacy students to work in a rural area is drastically lower than the NHSC student loan forgiveness program offered to other healthcare professionals. Specifically, over four years, pharmacy students would require roughly $75,000 to work in a rural area while the NHSC student loan program potentially pays new healthcare graduates $160,000 over that same period. This suggests that increasing salaries would be a more cost-effective way to improve recruitment instead of simply expanding the NHSC program to cover pharmacists, and the additional findings provide more insight into student job preferences. For example, the consistent insignificant results across all states for the ‘Community with 30%’ setting suggest that students are indifferent between these two different Community-based settings, though the same cannot be said when looking at hospital-based settings. Specifically, a hospital position with no patient interaction is found to be highly undesirable while one with some patient interaction is highly desirable. This is not overly surprising, given that only 54% of American pharmacy students go into community practice [20] compared to more than 70% of Canadian students [21], though it does suggest that nonfinancial incentives such as opportunities for advancement and more patient-interaction could be effective if they better align with student preferences [6,22].

### 4.2. Canada

Overall, Canadian pharmacy students seem to be relatively comparable to American students regarding their willingness to work in a rural setting, as they would require a similar $17,156 increase in salary to consider such a position. However, Canadian students seem relatively more willing to work in fringe settings, as they would require an additional $5198 in salary to do so compared to Americans needing $12,395. These findings are not overly surprising given the differences between these students and their preference for community practice, which are reinforced by the results regarding setting and patient interaction. Specifically, the consistently negative results for all settings (outside of hospital pharmacy with 30% patient interaction, which is statistically insignificant), suggest that community based-practice with significant patient interaction is the most preferred setting for graduating pharmacy students. Given this overwhelming preference, simultaneous efforts to both increase salaries and time spent with patients should effectively increase pharmacist recruitment to underserved areas. 

### 4.3. Differences in Student Preferences between States/Provinces

It is clear from Table 3 and Table 4 that student preferences and required financial incentive to practice in rural and more undesirable settings vary greatly. This may be due to culture of the state/province, influence of faculty at each institution, and where each college of pharmacy recruits their students. For example, colleges recruit from major urban centers across the United States, while it is common for Canadian students to attend a college of pharmacy that is nearest to their hometown. This illustrates that it is important to know what kinds of students attend which colleges in order to implement the most effective interventions. 

According to the Iowa findings, rural pharmacy positions may target recruitment activities to males and those students from a rural area. Past research has found similar findings in medical students. Female medical students are significantly less likely to take a position in a rural setting than their male counterparts [23]. It has been reported that female physicians fear professional isolation if they were to take a rural position [24]. It is unclear if these findings could be extrapolated to female pharmacy students. This is an area where further research could be beneficial. Hospital positions with very little patient interaction may be most desirable to males, P4 students, and those students from an urban area. Lastly, males and those from fringe hometowns prefer hospital positions with some patient interaction. 

In South Dakota, students from rural hometowns are more likely to prefer pharmacy positions in rural areas. This aligns with similar past research conducted in Australia [21,25]. Pharmacy positions in fringe areas are more appealing to students from a rural hometown. Lastly, employers with hospital positions with very little patient interaction may think about recruiting students from out of state. This is the least desired job setting for South Dakota students, requiring a significant financial incentive. However, students from other Midwest states are more commonly indifferent to this type of pharmacy setting.

Out of all the states and provinces studies, the province of Saskatchewan would have the best probability of recruiting any student to any pharmacy position. However, female students strongly prefer a community position with a lot of patient interaction to a community position with some patient interaction. 

In Manitoba, rural pharmacy positions are more likely to appeal to males and those students from a rural hometown. Males and P3 students are more likely to be indifferent to both hospital and community positions will very little patient interaction. 

### 4.4. Potential Interventions for Stakeholders

Based on the findings, below are suggested targeted recruitment efforts for stakeholders to engage in to recruit new graduates to locations that need qualified pharmacists. While post-graduate job placement is becoming more important, investigators strongly encourage equal hiring practices and in no way are embracing practices that would create a lack of diversity in a particular practice setting. 

Suggestions for Employers: Some employers in rural areas pay their pharmacists more per year in order to live and work in their area. However, this may not be enough. Efforts should focus on making up for the difference between employer compensation and required level of financial incentive. Employers should also become familiar with all the available federal and state student loan debt payoff programs for their location to highlight potential financial incentives to new graduates.

It is clear that pharmacy settings with higher levels of patient interaction were more desirable to students in the United States and Canada. Pharmacists practicing in medically underserved areas or areas with decreased health care access have the opportunity to become key health care professionals in the lives of their community [26,27]. Pharmacists practicing in rural areas report having high levels of patient interaction [28]. Employers or retiring pharmacy owners in rural areas should focus on highlighting the potential for higher patient interaction for positions in less desirable living areas. Canadian students preferred increased patient interaction significantly more than American students. 

Although these results help to provide more insight into recruitment strategies for rural pharmacies, it is unrealistic to believe that such pharmacies will be able to afford these higher salaries or sustain paying them over time. Because of this, loan reimbursement has become a more common practice and these results help to support its greater relative cost effectiveness as well. Specifically, the results for the full US sample suggest that pharmacy students would need to be paid an additional $18,738 in salary each year to consider working in a rural location; however, given that these same students are also willing to sacrifice $654 for every additional percentage point of loan repayment after five years, this $18,738 in salary would be perfectly offset by 28.65 percent of their loans being reimbursed after working for five years. With students attending private institutions again having roughly $193,296 in loans on average, paying 28.65 percent of those loans (or roughly $55,382) is significantly less expensive that paying $18,738 in additional salary each of those five years (or $93,690 in total).

Suggestions for Pharmacy Associations and Boards of Pharmacy: Connections are critical for a young professional. Pharmacy Associations and Boards of Pharmacy could make more efforts to utilize their membership list to identify pharmacist practicing in these rural and undesirable practice settings and connect these members with schools of pharmacies and student organizations to be potential resources for students. These pharmacists can guest lecture, assist in student programming/co-curricular events, or be identified as potential independent study/internship sites. By integrating pharmacists in these practice settings into the student educational experience, there is a potential to strike passion into a couple students and allow educators, academic advisors, and student services staff to identify what student may want to work in rural or more undesirable pharmacy settings. It is clear by the findings that some students are interested in working in these areas with little or no financial incentive. Therefore, identifying these students and aligning them with practitioners in those specific settings will help to grow the pharmacist pipeline in these areas. By leaning on their practitioner network, Pharmacy Associations and Boards of Pharmacy could become key players in addressing the workforce needs of all the pharmacies and communities they represent. 

Suggestions for Colleges of Pharmacy: It is apparent from the findings that students who were not from rural areas were less likely to prefer position openings in these areas. It is important that pharmacy students be exposed to working in a variety of settings during their education. It has been shown that when dental students were required to have a rural experiential experience, they were more likely to become indifferent to positions in rural areas [29]. Colleges of pharmacy may collaborate with rural hospitals and pharmacies to bring practitioners to campus so students can hear the students. This may be accomplished through guest lectures and student organization events. Ideally, this exposure opportunity would be integrated into a required course, as an internship opportunity, or experiential learning. Not only would this be a positive learning experience for all students, it would potentially allow for the identification of P3 students who are interested in or curious to learn more about practicing in rural areas. By identifying students earlier in their education, it is highly probable to “move” those students who are indifferent to preferring rural positions. This may lead to the rural employer to decreasing, and maybe eliminating, the financial incentive required. 

Some colleges of pharmacy have existing rural health initiatives. Here are three examples of such efforts. The University of North Carolina Earlman School of Pharmacy has a Rural Pharmacy Health Certificate for their pharmacy students that begins the Spring semester of their P1 year [30]. Drake University utilizes their relationship with other schools of health care professionals (Doctor of Osteopathic Medicine, Physical Therapy, and Physician Assistant) within the state of Iowa. Students can earn a Rural Health Initiative Certificate after completing five Saturdays throughout the academic year at various central Iowa locations [31]. One of the most in-depth student learning experience is the University of Chicago College of Pharmacy RPHARM Concentration. Not only does this program prepare students for pharmacy practice in a rural area, but also prepares students for rural life. In addition, students enrolled in the RPHARM are required to have half of their Advanced Pharmacy Practice Experiences (APPEs) in rural communities [32]. 

Alternatively, schools of pharmacy could identify one or two pharmacies in rural areas that are innovative and leading pharmacy service development. Schools can use these pharmacies as examples of practicing at the top of a pharmacy license in courses, co-curricular, or student programming. Alternatively, schools of pharmacy could identify a pharmacy in a rural area who may desire assistance to implement a pharmacy service they have had in mind, but may not have the time or human resources to implement it in a timely manner. Pharmacy students can then take on this entrepreneurial activity during a management course, co-curricular, independent study, or summer internship. By collaborating with the school of pharmacy (either through didactic work, co-curriculars, or experimental education), students can experience that type of working and living environment prior to applying for jobs.

### 4.5. Additional Factors Influencing Job Placement

Need for More and/or Better Student Professional Development: There have been arguments that the reason job placement post-graduation remains around 86% [19] is that some students lack the soft-skills, leadership, and innovation required at these locations [33]. Many rural community and hospital pharmacists have to remain innovative and entrepreneurial to provide quality patient care to their older, sicker, and geographically isolated communities [34]. Although ACPE Accreditation have standards that meet these qualities, this potentially highlights the need for schools of pharmacies to provide either more or higher quality professional development opportunities that align with what employers desire in a job candidate. 

Telepharmacy as an Alternative: While literature and news reports vary on if the pharmacy profession is experiencing growth or decline, in terms of number of practicing pharmacists, it is clear that there is a rapid decline of pharmacists working in rural areas [35]. This, along with the advancement of telemedicine delivery and reimbursement, has led to telepharmacy filling in those unfilled positions in rural or medically underserved areas in order for rural residents to maintain access to care. However, many challenges still exist in implementing telepharmacy [36]. Because telepharmacy utilizes state of the art technology in order to deliver care, start-up costs create a barrier to implementation [36]. In addition, some states lack legal definitions and regulation to support this model of practice. Therefore, it remains critical to recruit graduating students to these areas. 

## 5. Conclusions

This study utilized Discrete Choice Experiment methodology to quantify the exact financial incentive required for pharmacy students from the United States Midwest and Central Canada to accept a post-graduate position in rural areas or practice settings that have undesirable job traits. Pharmacy student preferences in post-graduation job attributes vary greatly between states and provinces. American and Canadian female students would require a larger financial incentive to take a position in a rural area or a job without a large amount of patient interaction. Overall, after adjusting for exchange rate, Canadian and American students would require, on average, a $17,156 and $18,738 to take a position in a rural area. In addition, other job characteristics such as time spent interacting with patients and opportunities for advancement have also been found to influence student preferences, suggesting that non-financial incentives may also be used to recruit pharmacy graduates to rural areas.

## Figures and Tables

**Table 1:** Operationalization of job attributes for economic valuation bundles.

Attribute Names	Attribute Levels
Geographic Location	Rural, Urban Fringe, Urban
Years to Promotion	1, 3, and 5 years to promotion
Practice Setting/Percent Patient Interaction	Community with 0% *, Community with 30%, Community with 70%, Hospital with 0%, Hospital with 30%
Salary	$75,000, $100,000, $115,000 (Canada $45/h, $50/h, $55/h)
Loan Repayment	0%, 10%, 30%, 100% (Canada—N/A)

* Due to a survey error, the ‘Community with 0%’ setting level was accidentally included in the survey administered in Manitoba.

**Table 2:** Overall and Subsample Subject Characteristics.

	US Full Sample n = 213 (%)	Canada Full Sample n = 70 (%)	Iowa n = 97 (%)	South Dakota n = 79 (%)	North Dakota n = 37 (%)	Saskatchewan n = 22 (%)	Manitoba n = 48 (%)
**Gender**							
Male	31.5	32.9	33	33	24.3	41	29.2
Female	68.5	67.1	67	67	75.7	59	70.8
**Year in School**							
P1	17	22.9	16.5	19	13.5	41	14.6
P2	22.5	20	18.7	29.1	19	18.2	20.8
P3	45	25.7	37.1	46.9	62.1	18.2	29.2
P4	13.5	31.4	27.8	5	5.4	22.6	35.4
**Hometown Population Size**							
Less than 2500	26.3	31.4	17.5	26.6	48.6	31.8	31.3
2500–25,000	37	31.4	35	39.2	37.9	27.3	33.3
25,000+	36.7	37.2	47.5	34.2	13.5	40.9	35.4

**Table 3:** Willingness to pay estimates of United States subjects (Reported in USD).

**United States (n = 213)**	**Full Sample**	**Male**	**Female**	**P1**	**P2**	**P3**	**P4**	**Rural**	**Fringe**	**Urban**
Location: Rural	−18,738 **	−19,653 **	−20,182 **	−14,979 **	−15,182 *	−21,423 **	−28,404 **	−2864	−33,010 **	−23,574 **
Location: Fringe	−12,395 **	−26,458 **	−6667	−4511	−10,848	−15,511 *	−8794	−13,394	−16,851 *	−5495
Years to Promotion	−2178 *	−1,94	−2139	−2617	−2185	−2270	979	−1124	−2782	−2291
Setting: Community with 30%	−5987	−3337	−9242*	4149	−23,424 **	−5438	−12,234	−11,394	−4429	−8949
Setting: Hospital with 0%	−17,023 **	−11,424	−19,818 **	−6681	−39,909 **	−15,876 *	2436	−26,364 **	−24,533 **	−8,138
Setting: Hospital with 30%	8544 *	9688	6727	7894	4758	6679	23,652	−3394	7,647	16,096 **
Loan Repayment (% Point)	654 **	722 **	600 **	509 **	733 **	686 **	571 **	706 **	644 **	577 **
**Iowa (n = 97)**	**Full Sample**	**Male**	**Female**	**P1**	**P2**	**P3**	**P4**	**Rural**	**Fringe**	**Urban**
Location: Rural	−25,143 **	−13,389	−35,177 **	−26,846 **	−86,845	−22,810 *	−28,404 **	−10,391	−31,762 **	−28,694 **
Location: Fringe	−15,102	−5017	−20,133	−6769	−155,428	−14,714	−8794	−32,913	−13,640	−10,000
Years to Promotion	201	−701	1465	1610	1032	122	979	2535	338	316
Setting: Community with 30%	−9429	−4551	−15,841	8026	−48,531	−18,619	−12,234	−10,783	−10,077	−13,510
Setting: Hospital with 0%	−16,898 *	−2854	−26,239 **	10,949	−25,705 **	−26,333 *	2436	−54,783 **	−22,452 *	−2665
Setting: Hospital with 30%	3886	21,993 *	−8363	16,949	−108,174	−8857	23,652	−22,217	4866	11,633
Loan Repayment (% Point)	653 **	565 **	686 **	390 **	2299 **	724 **	571 **	978 **	467 **	673 **
**North Dakota (n = 37)**	**Full Sample**	**Male**	**Female**	**P1**	**P2**	**P3**	**P4**	**Rural**	**Fringe**	**Urban**
Location: Rural	−8715	−10,855	−8242	−11,124	−2555	−8982	N/A	−5494	−11,589	−13,801
Location: Fringe	−11,465	−65,133 *	−3308	−8689	−8758	−6718		−43,210 *	−3282	1399
Years to Promotion	−2162	−1525	−2495	−3349	−2144	−3257		−4198	−684	−4637
Setting: Community with 30%	4473	−6490	4121	14,262	−5040	6260		−16,821	14,439	587
Setting: Hospital with 0%	1270	13,274	−2136	16,885	−1313	896		−4568	6114	−16,767
Setting: Hospital with 30%	13,368	2767	15,992 *	7799	19,870 *	10,000		12,531	24,819 *	−4968
Loan Repayment (% Point)	519 **	649 **	452 **	543 *	315 *	560 **		824 **	434 **	154
**South Dakota (n = 79)**	**Full Sample**	**Male**	**Female**	**P1**	**P2**	**P3**	**P4**	**Rural**	**Fringe**	**Urban**
Location: Rural	−13,942 **	−36,042 **	−11,701 *	−3861	−7807	−25,942 **	N/A	4957	−49,294 **	−14,498 *
Location: Fringe	−12,692 *	−37,257 *	−7908	−1554	−2988	−26,331		1911	−36,580 *	−9052
Years to Promotion	−3918 **	−4167	−3747 **	−4079 *	−2046	−3474		−777	−10,446 **	−3309
Setting: Community with 30%	−7380	−4653	−10,575	−2139	−27,229 **	−4318		−7447	−18,699	−5595
Setting: Hospital with 0%	−21,947 **	−41,215 **	−21,172 **	−20,059 **	−27,976 **	−17,922		−25,404 **	−53,941 **	−7045
Setting: Hospital with 30%	11,899 *	−7153	13,241 *	6733	12,337	17,922		−1117	−4424	24,851 **
Loan Repayment (% Point)	642 **	997 **	552 **	477 **	716 **	692 **		460 **	1030 **	528 **

* significant *p* = 0.05, ** *p* < 0.01.

**Table 4:** Willingness to pay estimates of Canadian subjects (Reported in USD).

**Canada (n = 70)**	**Full Sample**	**Male**	**Female**	**P1**	**P2**	**P3**	**P4**	**Rural**	**Fringe**	**Urban**
Location: Rural	−17,156 **	−19,224 **	−13,808 **	−2074	−5239 *	−26,543 **	−50,344 **	−14,860 **	−23,499 **	−14,815 **
Location: Fringe	−5198 **	−6361 *	−3368	4033	−4324	928	−15,836 **	−3829	−6081 *	−4627
Years to Promotion	−2149 **	−2304 **	−1697 **	−2185 **	−733	−2114	−1223	−2567 **	−1391	−1824 **
Setting: Community with 30%	−5277 **	−50	−7781 **	−2650	−1026	−19,444 **	−15,230 *	−7678 **	−6862 *	−505
Setting: Hospital with 0%	−10,410 **	−4839	−13,701 **	−8177	−5566 *	−22,568 **	−24,299 **	−15,606 **	−12,828 **	−3791
Setting: Hospital with 30%	1468	1476	513	1955	1799	471	−5152	−4484	−3664	10,227 *
Setting: Community with 0% ⧫	−18,306 **	−81,247	−18,690 **	−5218	−27,399	−158,530	−177,232	−81,732	−2562	−122,262
**Saskatchewan (n = 22)**	**Full Sample**	**Male**	**Female**	**P1**	**P2**	**P3**	**P4**	**Rural**	**Fringe**	**Urban**
Location: Rural	−7037	−11,739	−6322	610	−3025	31,022 *	N/A	−1917	17,184	1004
Location: Fringe	−1884	−3055	−2409	3107	4347	23,824		−2884	16,779	5579
Years to Promotion	−1519	−2071	−1504	−2244	1175	1824		−1944	4114	−470
Setting: Community with 30%	−6056	−202	−11,036 *	−2593	−9173 *	4324		−7601	−7426	−3939
Setting: Hospital with 0%	−10,457	−7921	−11,111	−6730	−17,371	−13,137		−14,375	−23,442	−9626
Setting: Hospital with 30%	−300	4699	−2484	−1732	−5047	−6381		−8821	767	3577
**Manitoba (n = 48)**	**Full Sample**	**Male**	**Female**	**P1**	**P2**	**P3**	**P4**	**Rural**	**Fringe**	**Urban**
Location: Rural	−22,173 **	−12,682	−20,086 **	−1934	−5794	60,265	−41,775 *	33,054	−18,687 *	−22,257 *
Location: Fringe	−4232	−9642	−1494	7907	−2835	−42,907	−12,566	−24,713	−7461	−4114
Years to Promotion	−2710 **	−1292	−2769 *	−2469	−626	7296	−2927	4799	−1474	−1876
Setting: Community with 30%	−7203 *	2238	−9195 *	−4286	−701	61,188 **	−9313	16,296	−3624	−6516
Setting: Hospital with 0%	−15,270 **	4350	−22,057 **	−14,815	−5741	88,330 **	−19,390	47,268	−8173	−13,479
Setting: Hospital with 30%	−2026	6860	−2145	2510	795	26,611	−8231	39,377	276	5204
Setting: Community with 0% ⧫	−22,734 *	644	−26,228 *	−8836	−37,860	486,093	−130,209	184,219	−381	−180,849

Note: ⧫ The surveys for Manitoba respondents accidentally included bundles that contained a community setting with 0% of the individual’s time spent interacting with patients, which is why this result is not available in other states/provinces; * significant *p* = 0.05, ** *p* < 0.01.

## Supplementary Materials

The following are available online at https://www.mdpi.com/2226-4787/7/3/109/s1, Table S1: Canadian Estimates in CAD.
