# Supplementary material for: Financial Incentive Required for Pharmacy Students to Accept a Post-Graduation Position in Rural and Undesirable Pharmacy Settings
Source: Pharmacy (Basel). 2019 Aug 6;7(3):109. doi: 10.3390/pharmacy7030109 (PMC6789724; doi:10.3390/pharmacy7030109)
Supplement: Supplementary file 1 [file pharmacy-07-00109-s001.pdf]

**Table S1.** Willingness to Pay Estimates for Canadian Subjects (Reported in Canadian Dollars).

| Canada (n = 70) | Full Sample | Male | Female | P1 | P2 | P3 | P4 | Rural | Fringe | Urban |
|---|---|---|---|---|---|---|---|---|---|---|
| Location: Rural | −23,161** | −25,952** | −18,641** | −2,800 | −7,073* | −35,833** | −67,965** | −20,061** | −31,723** | −20,000** |
| Location: Fringe | −7,018** | −8,587* | −4,547 | 5,444 | −5,837 | 1,253 | −21,378** | −5,169 | −8,209* | −6,247 |
| Years to Promotion | −2,902** | −3,111** | −2,291** | −2,950** | −989 | −2,854 | −1,651 | −3,466** | −1,878 | −2,462** |
| Setting: Community with 30% | −7,125** | −68 | −10,504** | −3,578 | −1,385 | −26,250** | −20,561* | −10,365** | −9,264* | −682 |
| Setting: Hospital with 0% | −14,054** | −6,532 | −18,496** | −11,039 | −7,514* | −30,467** | −32,804** | −21,068** | −17,318** | −5,118 |
| Setting: Hospital with 30% | 1,982 | 1,992 | 693 | 2,639 | 2,428 | 636 | −6,955 | −6,054 | −4,946 | 13,806* |
| Setting: Community with 0% | −24,714** | −109,683 | −25,231** | −7,044 | −36,989 | −214,015 | −239,263 | −110,338 | −3,459 | −165,054 |
| **Saskatchewan (n = 22)** | **Full Sample** | **Male** | **Female** | **P1** | **P2** | **P3** | **P4** | **Rural** | **Fringe** | **Urban** |
| Location: Rural | −9,500 | −15,847 | −8,535 | 824 | −4,084 | 41,880 | | −2,588 | 23,199 | 1,355 |
| Location: Fringe | −2,544 | −4,124 | −3,252 | 4,195 | 5,869 | 32162.00 | | −3,893 | 22,652 | 7,531 |
| Years to Promotion | −2,051 | −2,796 | −2,031 | −3,029 | 1,586 | 2,462 | n/a | −2,625 | 5,554 | −635 |
| Setting: Community with 30% | −8,176 | −273 | −14,898* | −3,500 | −12,384* | 5,838 | | −10,262 | −10,025 | −5,318 |
| Setting: Hospital with 0% | −14,118 | −10,693 | −15,000 | −9,086 | −23,451 | −17,735 | | −19,406 | −31,647 | −12,995 |
| Setting: Hospital with 30% | −406 | 6,343 | −3,354 | −2,338 | −6,814 | −8,615 | | −11,909 | 1,035 | 4,829 |
| **Manitoba (n = 48)** | **Full Sample** | **Male** | **Female** | **P1** | **P2** | **P3** | **P4** | **Rural** | **Fringe** | **Urban** |
| Location: Rural | −29,934** | −17,121 | −27,116** | −2,611 | −7,822 | 81,358 | −56,396* | 44,623 | −25,228* | −30,047* |
| Location: Fringe | −5,714 | −13,017 | −2,017 | 10,675 | −3,827 | −57,925 | −16,964 | −33,362 | −10,073 | −5,554 |
| Years to Promotion | −3,659** | −1,744 | −3,738* | −3,333 | −845 | 9,849 | −3,951 | 6,478 | −1,990 | −2,532 |
| Setting: Community with 30% | −9,725* | 3,021 | −12,413* | −5,786 | −947 | 82,604** | −12,572 | 22,000 | −4,893 | −8,797 |
| Setting: Hospital with 0% | −20,615** | 5,873 | −29,777** | −20,000 | −7,751 | 19,245** | −26,176 | 63,812 | −11,034 | −18,196 |
| Setting: Hospital with 30% | −2,736 | 9,261 | −2,896 | 3,389 | 1,073 | 35,925 | −11,112 | 53,159 | 372 | 7,025 |
| Setting: Community with 0% | −30,692* | 869 | −35,408* | −11,929 | −51,111 | 656,226 | −175,782 | 248,696 | −515 | −244,146 |

* significant $p = 0.05$, ** $p < 0.01$.
